# Cortical Sensorimotor Integration as a Neural Origin of Impaired Grip Force Direction Control following Stroke

**DOI:** 10.3390/brainsci14030253

**Published:** 2024-03-05

**Authors:** Christian Schranz, Na Jin Seo

**Affiliations:** 1 Department of Health Sciences and Research, College of Health Professions, Medical University of South Carolina, Charleston, SC 29425, USA; schranz@musc.edu; 2 Ralph H. Johnson VA Health Care System, Charleston, SC 20401, USA; 3 Department of Rehabilitation Sciences, College of Health Professions, Medical University of South Carolina, Charleston, SC 29425, USA

**Keywords:** stroke, upper extremity, rehabilitation, sensory afferent inhibition, sensorimotor integration, feedback motor control, TMS, paired associative stimulation

## Abstract

Background: Stroke is a major cause of disability worldwide. Upper limb impairment is prevalent after stroke. One of the post-stroke manifestations is impaired grip force directional control contributing to diminished abilities to grip and manipulate objects necessary for activities of daily living. The objective of this study was to investigate the neural origin of the impaired grip force direction control following stroke. Due to the importance of online adjustment of motor output based on sensory feedback, it was hypothesized that grip force direction control would be associated with cortical sensorimotor integration in stroke survivors. Methods: Ten chronic stroke survivors participated in this study. Cortical sensorimotor integration was quantified by short latency afferent inhibition (SAI), which represents the responsiveness of the primary motor cortex to somatosensory input. Grip force direction control was assessed during paretic grip. Results: Grip force direction control was significantly associated with SAI. This relationship was independent of sensory impairment level. Conclusions: Cortical sensorimotor integration may play a significant role in the grip force direction control important for gripping and manipulating objects with the affected hand following stroke. This knowledge may be used to inform personalized rehabilitation treatment. For example, for patients with impaired grip force direction control, behavioral therapy focusing on feedback motor control, augmented by use of brain stimulation to reinforce cortical sensorimotor integration such as paired associative stimulation, may be applied.

## 1. Introduction

Stroke is the leading cause of long-term disability worldwide [1]. Approximately 80% of stroke survivors suffer from persistent impairment in hand function, even after completing a full course of rehabilitation services [2]. Hand impairment diminishes stroke survivors’ abilities to perform the meaningful activities of daily living and thus lowers their functional independence and quality of life [3].

One primary function of the hand is gripping and manipulating objects for activities of daily living. Biomechanically, it is essential to generate adequate grip force in the precise direction to successfully pick up and manipulate an object [4,5,6,7]. When grasping an object with the thumb and index finger or in cylindrical grip, for example, the horizontal forces should counterbalance each other, and failure to do so would unbalance the grasped object, resulting in unwanted rotations of the object [4,5,8]. Furthermore, when the digit force is applied with a substantial shear force, such that the ratio of shear force to normal force exceeds the coefficient of friction between the fingertip pad and grip surface, the finger slips against the grip surface and loses the grip of the object [5,6,7,9]. The coefficient of friction for the fingertip pad skin has been shown to be not different between stroke survivors and age-matched controls [10]. Thus, the same slip threshold applies for both paretic and nonparetic fingers.

**Figure 1:**
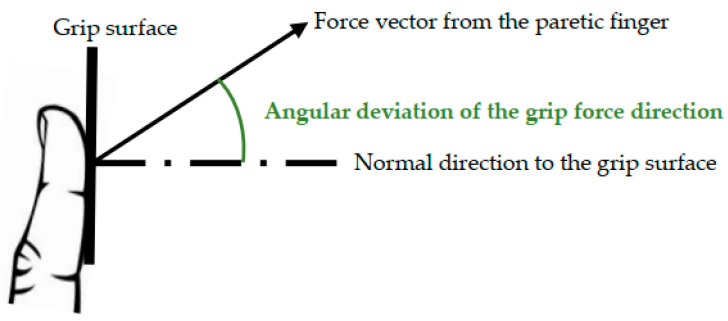
Illustration for grip force direction control.

Unfortunately, one manifestation of upper limb impairment post stroke is the diminished ability to apply digit force in the proper direction per task demand (Figure 1) [7]. It was found that the deviation of the grip force direction from the direction normal to the grip surface was significantly greater for the paretic hand compared to the nonparetic hand of chronic stroke survivors, as well as age-matched, neurologically intact adults [11]. This greater deviation of the grip force direction in stroke survivors was observed for both the thumb and index fingers, regardless of the object’s size [11]. The greater deviation of the grip force direction in the paretic hand was observed during grasp of not only an unconstrained object but also a stationary object (i.e., an object freely moving with the hand vs. an object fixed to a table) [11]. It was observed for the precision grip with the thumb and index finger [11] as well as the cylindrical grip in all segments of the digit in stroke survivors [12]. It was observed regardless of the grip force level [11,12].

The consequence of this impaired grip force direction control is an impaired ability to grasp and manipulate objects for activities of daily living [7]. The impaired grip force direction control was accompanied by the paretic fingers slipping, moving greater than 1 cm during over half of all gripping trials, while no slipping was observed for the nonparetic fingers as measured by a three-dimensional motion capture system [11]. This finger slip presents a hindrance to successfully gripping and manipulating an object. As such, the extent of impaired grip force direction control was found to be significantly associated with a manual dexterity measure of the Box and Block Test [7]. Interestingly, although all stroke survivors in the study had the capacity to produce the grip force magnitude necessary to lift the block against gravity, among those who could open their hand, the Box and Block Test score (i.e., the number of blocks that could be moved in 1 min) varied substantially depending on the extent of deviation of their grip force direction from the normal direction [7]. The extent of impaired grip force direction control was also found to be significantly associated with the upper extremity impairment scores of the Fugl-Meyer Assessment of Motor Recovery after Stroke [13,14] Hand/Wrist section and the Chedoke–McMaster Stroke Assessment [15,16] Hand Stage in the previous study [7]. This finding illustrates the importance of grip force direction control in hand function.

As for the cause of the impaired grip force direction control following stroke, previous research has shown that it arose from altered paretic muscle activation patterns [11,12,17,18], rather than from postural disparities [11]. In addition, previous research has shown that stroke survivors with tactile sensory deficit exhibit greater impairment in the grip force direction control than stroke survivors without tactile sensory deficit [18], which is consistent with literature on the importance of sensory feedback for grip control [19]. However, the responsible processes in the central nervous system for grip force direction control have not been investigated in stroke survivors to date.

We hypothesize that sensorimotor integration may be a process in the central nervous system that may be responsible for the grip force direction control following stroke. Stroke survivors with impaired grip force direction control may grasp an object with excessive shear force, due to the lack of incorporation of sensory information about the grip force direction from mechanoreceptors in the finger skin detecting skin deformation and microslipping [7,18,20,21]. In other words, the grip force direction control may require sensorimotor integration, i.e., online adjustment of motor output based on sensory feedback necessary for feedback motor control [22,23].

One method to assess sensorimotor integration is the short latency afferent inhibition (SAI) [24]. SAI is assessed within the paired associative stimulation paradigm as the percent decrease of the single-pulse transcranial magnetic stimulation (TMS)-induced motor evoked potential (MEP) amplitude when TMS is preceded by a single-pulse electrical median nerve stimulation at the wrist 20–30 ms prior [25]. SAI is known to be mediated at the level of the motor cortex [25] via suppression of I2 and I3 waves [26] through cholinergic circuits [27], which is necessary for experience-dependent plasticity [28,29]. Thus, SAI represents responsiveness of the primary motor cortex (M1) to sensory input from the primary sensory cortex (S1) [30].

In stroke survivors, SAI has been implicated as a potential marker for motor recovery [24,31,32]. Specifically, reduction in SAI in the acute phase after stroke was associated with 6-month functional status [31]. In the chronic phase, reduced SAI related to greater motor impairment [32]. An increase in SAI with intervention was associated with improved motor function in the chronic phase [24]. These findings highlight the potential clinical relevance of SAI in stroke survivors. However, the cross-sectional relationship between SAI and grip force direction control has not yet been investigated.

Toward this end, the objective of this study was to investigate the neural origin of the impaired grip force direction control post stroke. Specifically, we examined if the extent of impaired grip force direction control in stroke survivors is explained by the extent of impaired cortical sensorimotor integration. SAI was used as a relevant neurophysiologic biomarker for the cortical sensorimotor integration in this study.

## 2. Materials and Methods

### 2.1. Participants

For this pilot study, a convenience sample of ten stroke survivors with a presence of MEPs from the ipsilesional hemisphere were included. They were chronic stroke survivors who suffered a stroke more than 6 months ago. A summary of the demographic and clinical characteristics of the participants is provided in Table 1. The average age of the ten participants was 61 years of age (standard deviation, SD = 11), and the average time since the last stroke was 5.0 years (SD = 3.2). There were an equal number of male and female participants. Their upper extremity impairment level as measured by the Fugl-Meyer Assessment of Motor Recovery after Stroke [13,14] ranged from 25 to 62 out of the highest possible score of 66. This score range represents a mild to moderate impairment level [33]. The upper extremity manual dexterity as measured by the Box and Block Test [34] ranged from 4 to 53. The Box and Block Test scores the number of blocks a person can move from one box to another with one hand in one minute, and its reliability and validity have been demonstrated in the literature [34]. The lower bound of this score indicates arduous effort needed to grasp and move objects, whereas the higher bound of this score indicates a manual dexterity level just outside of 2 standard deviations from the normative score [35]. Their pressure sensing abilities on fingertips as measured by the Semmes–Weinstein Monofilament test ranged from 2.36 to 4.17 in the monofilament size (corresponding to grams force of 0.02 to 1.4). This range includes normal sensation, diminished light touch, and diminished protective sensation [36]. Their spatial sensing resolution as measured by the two-point discrimination test ranged from 2 to 15 mm (by which the separation of two prongs could be perceived by a person) [36]. None of the participants had botulinum toxin injections in the upper limb within 3 months of participation in this study. The study protocol was reviewed and approved by the local Institutional Review Board prior to conducting this study. A written informed consent was obtained from all participants prior to their participation.

### 2.2. Brain MRI

**Figure 2:**
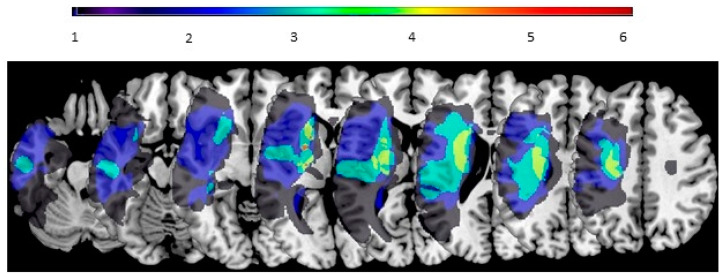
Lesion locations of participants with the affected hemisphere on the left side and the unaffected hemisphere on the right. The number of participants is represented in the color scale.

To facilitate navigation of the TMS application for participants with stroke, individual participants’ brain MRI was obtained. Specifically, a structural T1 weighted brain MRI was acquired using the MPRAGE sequence [37] on a Siemens Prisma 3T TIM Trio MRI scanner (Siemens AG, Munich, Germany) with an isometric 1 cubic mm voxel size. The dicom files from the scanner were converted to the NIfTI format showing the brain image in three-dimensions using MRIcro v1.0.2. [38]. Individual participants’ lesions were assessed following the established method [39,40]. A summary of the lesion locations of the participants is provided in Figure 2.

### 2.3. Short Latency Afferent Inhibition (SAI) Measures

The data collection session for SAI was conducted while participants were seated and resting (Figure 2). SAI was assessed using TMS and median nerve stimulation [41]. TMS was applied to the affected hemisphere using a Magstim^®^ 200^2^ stimulator with a 70 mm figure-of-eight coil (The Magstim Company Limited, Whitland, UK) held in a posterior–anterior direction. Surface electromyography (EMG) of the contralateral abductor pollicis brevis (APB) muscle in a belly-tendon montage (Figure 2) was collected to record the MEP induced by TMS. The EMG was collected through a CED 1902 Signal conditioner and 1401 interface (Cambridge Electronic Design, Cambridge, UK) with a sampling frequency of 5 kHz in Spike 2 software (Cambridge Electronic Design Ltd., Cambridge, UK).

Brainsight neuronavigation software v2.5.1. (Rogue Research Inc., Montreal, QC, Canada) was used to support the TMS application for the participants. The MRI brain image was imported into the Brainsight neuronavigation software. The brain image was reconstructed to show the cortical surface and co-registered with the anatomical landmarks of the tip of the nose, nasion, right and left outer canti of the eye, and right and left ear.

**Figure 3:**
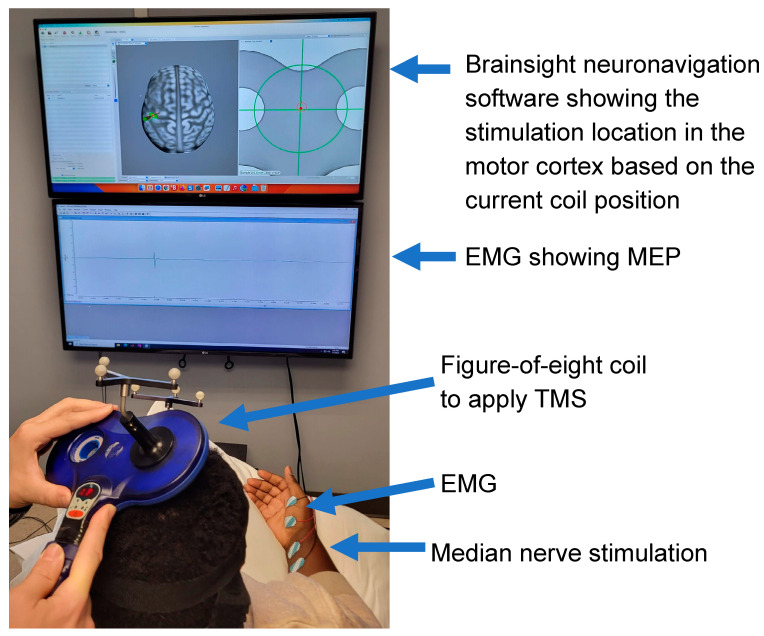
Measurement setup for the short latency afferent inhibition (SAI) including transcranial magnetic stimulation (TMS), median nerve stimulation, and electromyogram (EMG).

First, the hotspot for the APB muscle in the affected hemisphere was identified. The hotspot is defined as the location on the motor cortex that has neurons with monosynaptic projections to the alpha motor neuron in the spinal cord [42]. During TMS application, the Brainsight neuronavigation software displayed the locations of the TMS coil on the individual participant’s cortex, as shown in Figure 3, to guide the exploration of the cortex in search of the hotspot. The location that resulted in the largest MEP was determined as the hotspot.

Second, the resting motor threshold was determined following the guidelines of the International Federation of Clinical Neurophysiology [42]. Specifically, the resting motor threshold is defined as the percentage of the maximum stimulator output needed to elicit at least 0.05 mV peak-to-peak MEP amplitude (to assess the presence or absence of MEP) on the resting muscle with a 50% probability. The maximum-likelihood strategy for Parameter Estimation by Sequential Testing (PEST) [43] was used. The resting motor threshold ranged from 31% to 94% (mean 49%) of the maximum stimulation output across participants.

Next, the TMS recruitment curve [44] was obtained. For the recruitment curve, the starting stimulation intensity used was 80% of the resting motor threshold. The maximum stimulation intensity used for the recruitment curve was 3 times that of the resting motor threshold or 95% of the maximum stimulator output, whichever was less. The stimulation intensity changed in steps of 5% maximum stimulation output. MEP was obtained 6 times for each intensity. The TMS intensity used for SAI measurement was set to the level that resulted in 50% of the maximum peak to peak MEP based on the TMS recruitment curve. This level was chosen to maintain sensitivity to both the increase and decrease in the MEP amplitude by the conditioning electrical nerve stimulation. This approach is also consistent with previous literature [24].

The conditioning electrical nerve stimulation was delivered using a constant current generator (Constant Current DS7A, Digitimer Ltd., Hertfordshire, UK). Surface electrodes were placed on the wrist over the median nerve with the cathode proximal (Figure 2) to elicit median nerve stimulation [45]. A 0.2 ms duration electrical square pulse was used. The intensity of the electrical nerve stimulation to be used for SAI measurement was set to be three times the sensory threshold.

The sensory threshold for the electrical nerve stimulation was determined using the staircase method [46]. In short, the stimulation intensity was increased until the participant could perceive the stimulation (denoting the ascending threshold) and then decreased until the participant could not perceive the stimulation any longer (denoting the descending threshold). This procedure was repeated 3 times until 3 ascending and 3 descending thresholds were obtained. The average of the 6 thresholds was used as the sensory threshold.

Interstimulus intervals between the electrical nerve stimulation and TMS of 20, 25, 30 and 40 ms were used [24]. These intervals were chosen to include the interval that typically evokes SAI in healthy adults (20–25 ms) [25,47] and also longer intervals to account for interindividual variability [48] and the possible neural delay that may be present in stroke survivors [24]. The interstimulus interval was controlled in precision by the DG2A Train/Delay generator (Digitimer Ltd., Hertfordshire, UK).

**Figure 4:**
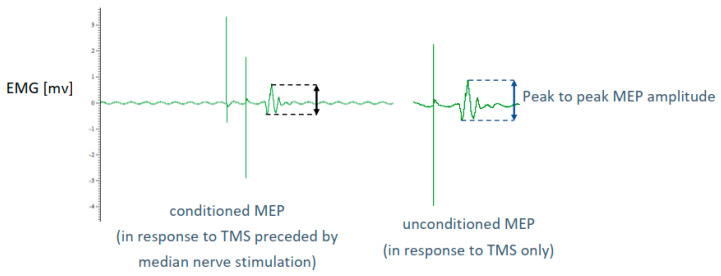
Illustration of a conditioned (**left**) and an unconditioned (**right**) motor evoked potential (MEP).

Conditioned MEPs at each interstimulus interval and unconditioned MEPs were obtained 16 times each to obtain an average. MEPs were visually inspected for quality. A representative conditioned and unconditioned MEP are shown in Figure 4. The order of obtaining the conditioned MEPs of different interstimulus intervals and non-conditioned MEPs were randomized in blocks for each participant. During TMS application, the Brainsight neuronavigation software displayed the location of the TMS coil relative to the hotspot to assist with maintaining the TMS coil location including its angle to ensure the application of TMS to the hotspot location.

SAI was computed as 1 minus the ratio of the conditioned peak to peak MEP amplitude (in response to TMS preceded by the median nerve stimulation) to the unconditioned peak to peak MEP (after TMS only without the median nerve stimulation) (1) [49]. The mean SAI was calculated for each interstimulus interval. The interstimulus interval that resulted in the highest mean SAI was determined for each participant. The corresponding SAI for that interstimulus interval was used for further analysis. This choice followed the previously published protocol that considered possible neural delay and interindividual variability in stroke survivors [24].

SAI = 1 − conditioned peak to peak MEP/unconditioned peak to peak MEP(1)

### 2.4. Grip Force Direction Control Measure

**Figure 5:**
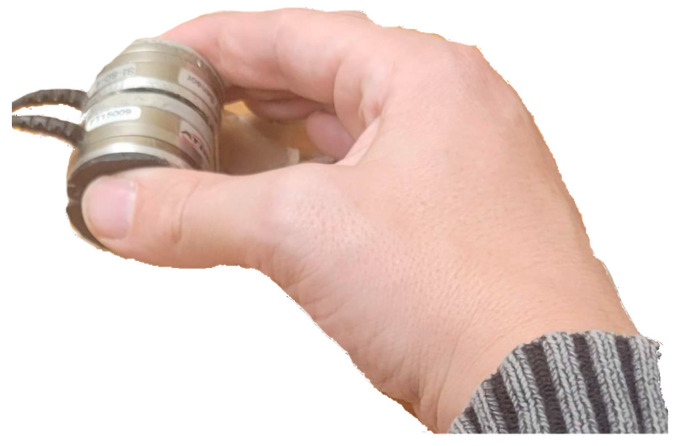
Setup for the grip force direction control measure.

**Figure 6:**
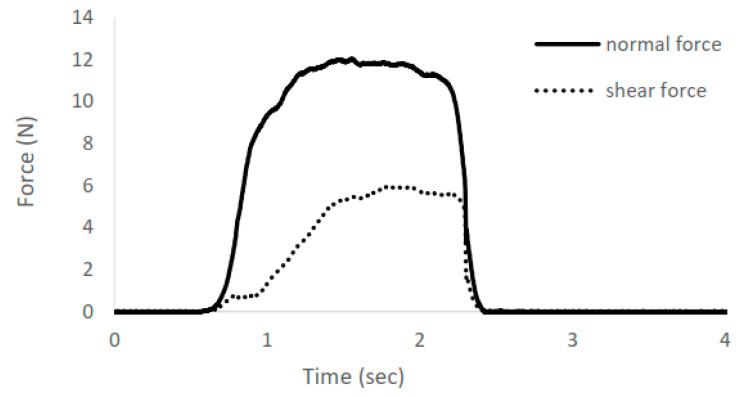
An example of grip force decomposed in normal force (in the direction normal to the grip surface) and shear force (in the direction tangential to the grip surface, square root of the sum of squared shear forces in two shear directions) over time. The larger shear force relative to the normal force represents a greater angular deviation of the digit force from the direction normal to the grip surface and thus worse grip force direction control.

For measurement of grip force direction control, the participants were instructed to grip an instrumented object using the thumb and index finger for a few seconds (Figure 5). A stationary object was used to grasp because previous research shows that stroke survivors exhibit impaired grip force direction control during grasping of both unconstrained and stationary objects [11]. The grasped object was instrumented with 6-axis load cells (Mini40, ATI Industrial Automation Inc., Apex, NC, USA) to record the force from the fingertips in 3 dimensions (Figure 6). Grip force direction was computed as the angular deviation of the force from the fingertip from the direction orthogonal to the grip surface (i.e., arc tangent of the ratio of shear force to normal force), averaged for the two fingers [7].

### 2.5. Statistical Analysis

The role of the cortical sensorimotor integration in grip force direction control was investigated by assessing the relationship between SAI and grip force direction control using regression. Both SAI and grip force direction control data were normally distributed (with a Shapiro–Wilk normality test *p* > 0.05), and thus, a Pearson correlation was used. As secondary analysis, the influence of sensory impairment was also investigated using regression. The sensory impairment data were not normally distributed, and thus, a Spearman’s rank-order correlation was used for this analysis. SPSS statistical software 29.0 (IBM Corp., Armonk, NY, USA) was used for statistical analysis.

## 3. Results

**Figure 7:**
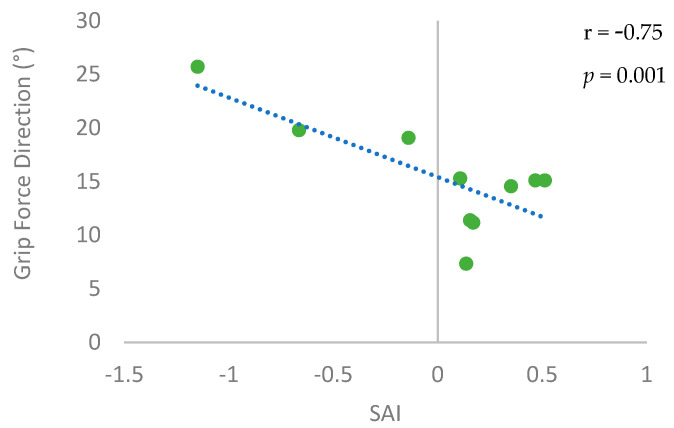
Association between the grip force direction control and the short latency afferent inhibition (SAI). Each participant’s data is shown as a dot. The fitted regression line is shown as a dotted line.

SAI was significantly associated with grip force direction control (Figure 7). The correlation coefficient was high (r = −0.75) and statistically significant (*p* = 0.01). Participants with higher SAI (i.e., higher afferent inhibition) had a lower deviation of the grip force direction from the normal direction (i.e., better control). Interestingly, the level of sensory impairment as measured by the Semmes–Weinstein Monofilament test and the two-point discrimination test were not significantly associated with SAI or grip force direction control (|r| ≤ 0.37, *p* ≥ 0.29).

## 4. Discussion

This pilot study provides evidence that the ability to control grip force direction may be associated with cortical sensorimotor integration as measured by SAI in chronic stroke survivors. Specifically, stroke survivors with higher level of SAI (i.e., greater inhibition of MEP by sensory input) tended to apply their grip force closer to the direction normal to the grip surface. In other words, stroke survivors with a higher level of cortical sensorimotor integration are more likely to exhibit better grip force direction control. This finding suggests that cortical sensorimotor integration may be a neural origin of impaired grip force direction control following stroke. The grip force direction control is directly associated with the manual dexterity and upper extremity impairment level in stroke survivors [7].

### 4.1. The Role of SAI for Functional Recovery Post Stroke

The finding of this study complements previous research investigating SAI following stroke. While there is not an extensive body of literature investigating SAI in stroke survivors [50], a few previous studies have shown that SAI is reduced in the stroke-affected side, compared to neurotypical adults or the unaffected side of stroke survivors, in both the acute [31] and chronic phase [24]. Patients with reduced SAI in the acute phase after stroke were found to be more likely to have better functional independence as measured by the modified Rankin Scale at 6 months post stroke, among 16 stroke survivors examined [31]. While SAI was found to be a significant factor for functional independence at 6 months, other corticomotor excitability measures, such as the resting motor threshold, active motor threshold, MEP amplitudes, and short interval intracortical inhibition, were not significantly associated with the 6-month functional independence score [31]. These results support the relevance of SAI for functional outcomes in stroke survivors. This research also suggests that the reduction in SAI after a stroke may represent a potential for motor recovery involving disinhibition of the central inhibitory circuits [51], long-term potentiation of M1, and increased synaptic efficiency for motor relearning [31,52].

Interestingly, in a rehabilitative intervention study, the opposite trend was observed in which performance improvement was accompanied by increased SAI [24]. Specifically, application of intermittent theta burst stimulation to the ipsilesional primary motor cortex followed by dexterity training led to a transient improvement in the time required for the paretic hand to lift an object upon grasping, which was accompanied by an increase in SAI in 13 chronic stroke survivors [24]. Similarly, in 15 healthy young adults who underwent a maze tracing practice, those with increases in SAI tended to have larger improvements in their speed and accuracy of the maze tracing task [53]. Therefore, while initial reduction in SAI of stroke survivors in the acute phase may represent a potential for neuroplasticity, rehabilitative training or task practices appear to induce an increase in SAI or restoration of SAI. A similar trend has been shown for the short intracortical inhibition in the affected hemisphere in which disinhibition early after stroke was followed by restoration of inhibition [54].

The cross-sectional relationship between SAI and upper limb functional performance has also been examined. Among 24 healthy young adults, it was observed that SAI was not significantly associated with the level of performance in the temporal order judgment task, grating orientation task, and pegboard test [55]. It is possible that the variation of SAI or task performance level among healthy young adults was too small to result in such a relationship. In 14 chronic stroke survivors, decreased SAI was found to relate to greater motor impairment [32]. Consistently, the new finding provided in the present study indicates that stroke survivors with greater SAI may have better grip force direction control. These stroke survivors were in the chronic stage (>6 months post stroke). Thus, the level of SAI in the chronic stage may indicate the state of restoration of SAI and function after rehabilitative treatments.

### 4.2. The Neural Origin of Impaired Grip Force Direction Control Post Stroke

The results of this study also expand the findings of previous research investigating the neural correlates of the grip force direction control following stroke. Previous studies have examined the connectivity of the brain’s neural circuits as associated with the grip force direction control following stroke [56,57]. For example, one study used diffusion tensor imaging (DTI) to assess the structural connectivity of the cortical and subcortical areas of the brain in 22 chronic stroke survivors [57]. This study found that poor grip force direction control was associated with lower structural connectivity in the network involving the bilateral Rolandic, ipsilesional supplementary motor area and contralesional thalamus [57]. Another study used a different approach using electroencephalography (EEG) in 12 chronic stroke survivors [56]. They found that poor grip force direction control was associated with lower functional neural connectivity between the contralesional premotor and primary somatosensory cortices [56].

The present study complements these previous studies and provides new evidence using the approach of paired stimulation using TMS and median nerve electrical stimulation. The present study suggests that cortical sensorimotor integration may be a potential biomarker for grip force direction control following stroke. We surmise that impaired performance in grip force direction control in the hand affected by a stroke may be manifested as a result of impaired cortical sensorimotor integration. We postulate that the performance may be facilitated by utilizing the residual neural resources including the sensorimotor network in the non-lesioned hemisphere as suggested by the previous studies [56,57].

In this study, SAI was negative, indicating a lack of inhibition, in 3 out of 10 participants. However, the presence versus lack of inhibition was not clearly associated with the lesion locations. Similarly, two previous case reports provide conflicting results that SAI was found abolished in a person with a thalamic lesion [58] and intact in another person with a thalamic lesion [59]. Future research with a larger sample size may be needed to investigate the role of different brain regions for SAI.

### 4.3. Influence of Sensory Impairment on SAI and Grip Force Direction Control Post Stroke

Interestingly, the sensory impairment level was not significantly associated with SAI nor grip force direction control in this cohort of stroke survivors. Previous studies also observed a lack of SAI in the presence of normal somatosensory evoked potential in a stroke survivor with a thalamic lesion [58] and a presence of SAI in the absence of N20 component of the somatosensory evoked potential in another stroke survivor [59]. These data suggest that SAI and sensory processing may be independent processes. While the present study did not show evidence that the sensory impairment level affects the grip force direction control, the present study cohort did not include those with severe sensory impairment such as those with a loss of protective sensation. Previous literature has shown evidence that sensory impairment may influence grip force direction control in the cylindrical grip of stroke survivors when comparing those with sensory deficit and those without [18]. Furthermore, sensory impairment is known to significantly hinder motor learning [60] and thus reduces the responsiveness to rehabilitation treatment [61] and impedes motor recovery [62,63]. Thus, the influence of sensory impairment may warrant further investigation with a larger sample size and a wide range of sensory impairment levels.

### 4.4. Clinical Implications

Previously, corticospinal integrity has been linked to upper limb impairment in both the subacute [64] and chronic phases after stroke [65,66]. This present study contributes to improved understanding of neural mechanisms behind impaired hand function post stroke by investigating the relationship between cortical sensorimotor integration and grip force direction control. Specifically, this research hints that cortical sensorimotor integration may be implicated for impaired grip force direction control in stroke survivors. Further strengthening the evidence on the relationship between sensorimotor integration and hand function in future research is expected to pave the way for the development of novel treatments [67] directly targeting sensorimotor integration. Many current intervention approaches are primarily focused on addressing the corticomotor pathway [68] with limited consideration of sensorimotor integration [69]. The present study suggests that restoration of effective sensorimotor interaction within the damaged motor system may be crucial for motor recovery [22]. For example, rehabilitation treatment for stroke survivors with poor grip force direction control and dexterity issues may focus on practicing tasks that utilize sensorimotor integration, such as feedback motor control [69]. It may also include the use of biofeedback of the grip force direction for explicit feedback [70]. Such targeted manual therapy may be augmented by the use of a non-invasive neuro-modulatory technique such as paired associative stimulation [71], as opposed to repetitive TMS targeting the corticospinal pathway [68]. Once the role of SAI is established by higher powered follow-up research, SAI may serve as a neurologic biomarker for future interventions targeting sensorimotor integration in stroke survivors [24,71]. Such development of novel treatments may address the impaired grip force direction and thus improve stroke survivors’ hand grip function [7].

### 4.5. Limitations and Future Direction

There are several limitations in the present research. First, in this study, we followed a previous protocol [24] and selected the SAI measures from the ISI that produced the highest inhibition to account for possible neural delay and interindividual variability in stroke survivors and thereby provide clinically meaningful results. The use of different ISI may have affected SAI measurements. Research in healthy individuals has shown that SAI can be obtained with moderate reliability with an ISI of 20–25 ms [72,73], and at higher ISI, there may be facilitation [47]. In our study, however, two participants had positive SAI (inhibition) only at an ISI of 30 ms, while having negative SAI (facilitation) at 20, 25, and 40 ms. This could be due to a delay in the afferent signal reaching the cortex as seen by the latency of N20 sensory evoked potentials as high as 25 ms in some stroke survivors [74]. It also suggests that the recommendations based on data from healthy individuals may not directly carry over to stroke survivors as postulated in previous studies [45,72]. Yet, the ISI could have been better individualized by using the latency of N20 sensory evoked potential +2–5 ms [31,45,47], which is the limitation of the present study. Second, this study was based on a limited sample of 10 stroke survivors. Unfortunately, the other previous studies investigating SAI in stroke survivors also had small sample sizes of 16, 14 and 13 [24,31,32]. Therefore, the result in the present paper along with the existing literature regarding the role of SAI in post-stroke motor recovery should be considered preliminary in nature and interpreted with caution. Follow-up research with a larger sample size is needed to investigate the role of SAI in post-stroke motor recovery with greater statistical power and with potential subgroup analysis.

This future analysis may include additional considerations such as sensory impairment, lesion locations, short and long latency afferent inhibition, motor state (resting versus movement) [49], and different muscles of the hand as multiple muscles’ coordination is critical for grip force direction control [11,17,75] and post-stroke impairment differs by muscle groups [76,77]. In addition, both lesioned and non-lesioned hemispheres may be investigated with a secondary analysis regarding the impact of the dominant versus non-dominant side on SAI. In healthy individuals, SAI magnitude has been reported to be greater in the dominant side than the non-dominant side [78], but this remains to be investigated in stroke survivors with additional consideration of the affected side. Additionally, while this study presented data from stroke survivors with a presence of MEP, MEP is not always present in stroke survivors [79]. Investigation on sensorimotor integration in these individuals without MEP is not possible with SAI and may require an alternative approach.

## 5. Conclusions

This research explored the neural origin of grip force direction. It examined the association between grip force direction and SAI, a measure of cortical sensorimotor integration. The results indicate that impaired grip force direction may be explained by impairment of sensorimotor integration. Furthermore, it suggests that sensory processing may be independent of SAI. Due to the small sample size in this study, additional research is necessary to ensure that these findings persist in a larger sample of stroke survivors. The knowledge of the neural origin of impaired grip force direction control may guide the development of novel treatment strategies that directly target the relevant neural circuit for optimal rehabilitation results.

## Figures and Tables

**Table 1:** Participant characteristics including demographic information and functional status.

Characteristics	Descriptive Statistics
Age in years (mean ± SD)	61.1 ± 10.9
Gender (male/female)	5/5
Race (White/African American)	5/5
Type of stroke (ischemic/hemorrhagic)	8/2
Affected side (right/left)	7/3
Time since stroke in months (mean ± SD)	59.9 ± 38.4
Fugl-Meyer Assessment of Motor Recovery after Stroke—Upper Extremity score (mean ± SD out of 66)	47.8 ± 11.6
Box and Block Test score (# of blocks that could be moved with the paretic hand in one minute, mean ± SD)	30.0 ± 16.9

## Data Availability

Deidentified data will be made available upon reasonable request. The data are not publicly available due to concerns about data misuse.

## References

[1] Katan M., Luft A. (2018). Global Burden of Stroke. Semin. Neurol..

[2] Lawrence E.S., Coshall C., Dundas R., Stewart J., Rudd A.G., Howard R., Wolfe C.D. (2001). Estimates of the prevalence of acute stroke impairments and disability in a multiethnic population. Stroke.

[3] Stewart J.C., Cramer S.C. (2013). Patient-reported measures provide unique insights into motor function after stroke. Stroke.

[4] Soechting J.F., Flanders M. (2008). Sensorimotor control of contact force. Curr. Opin. Neurobiol..

[5] Fikes T.G., Klatzky R.L., Lederman S.J. (1994). Effects of Object Texture on Precontact Movement Time in Human Prehension. J. Mot. Behav..

[6] MacKenzie C.L., Iberall T. (1994). The Grasping Hand.

[7] Seo N.J., Enders L.R., Motawar B., Kosmopoulos M., Fathi-Firoozabad M. (2015). The extent of altered digit force direction correlates with clinical upper extremity impairment in chronic stroke survivors. J. Biomech..

[8] Enders L.R., Seo N.J. (2011). Phalanx force magnitude and trajectory deviation increased during power grip with an increased coefficient of friction at the hand-object interface. J. Biomech..

[9] Seo N.J., Armstrong T.J., Drinkaus P. (2009). A comparison of two methods of measuring static coefficient of friction at low normal forces: A pilot study. Ergonomics.

[10] Hermsdorfer J., Hagl E., Nowak D.A., Marquardt C. (2003). Grip force control during object manipulation in cerebral stroke. Clin. Neurophysiol..

[11] Seo N.J., Rymer W.Z., Kamper D.G. (2010). Altered digit force direction during pinch grip following stroke. Exp. Brain Res..

[12] Enders L.R., Seo N.J. (2015). Altered phalanx force direction during power grip following stroke. Exp. Brain Res..

[13] Fugl-Meyer A.R., Jääskö L., Leyman I., Olsson S., Steglind S. (1975). The post-stroke hemiplegic patient. 1. a method for evaluation of physical performance. Scand. J. Rehabil. Med..

[14] Gladstone D.J., Danells C.J., Black S.E. (2002). The fugl-meyer assessment of motor recovery after stroke: A critical review of its measurement properties. Neurorehabilit. Neural Repair.

[15] Miller P., Huijbregts M., Gowland C., Barreca S., Torresin W., Moreland J., Dunkley M., Griffiths J., Van Hulenaar S., Vanspall B. (2008). CHEDOKE-McMASTER Stroke Assessment: Development, Validation, and Administration Manual.

[16] Gowland C., Stratford P., Ward M., Moreland J., Torresin W., Van Hullenaar S., Sanford J., Barreca S., Vanspall B., Plews N. (1993). Measuring physical impairment and disability with the Chedoke-McMaster Stroke Assessment. Stroke.

[17] Seo N.J., Fischer H.W., Bogey R.A., Rymer W.Z., Kamper D.G. (2011). Use of visual force feedback to improve digit force direction during pinch grip in persons with stroke: A pilot study. Arch. Phys. Med. Rehabil..

[18] Enders L.R., Seo N.J. (2017). Effects of Sensory Deficit on Phalanx Force Deviation During Power Grip Post Stroke. J. Mot. Behav..

[19] Nowak D.A., Hermsdorfer J. (2005). Grip force behavior during object manipulation in neurological disorders: Toward an objective evaluation of manual performance deficits. Mov. Disord..

[20] Parry R., Soria S.M., Pradat-Diehl P., Marchand-Pauvert V., Jarrassé N., Roby-Brami A. (2019). Effects of Hand Configuration on the Grasping, Holding, and Placement of an Instrumented Object in Patients With Hemiparesis. Front. Neurol..

[21] Westling G., Johansson R.S. (1984). Factors influencing the force control during precision grip. Exp. Brain Res..

[22] Bolognini N., Russo C., Edwards D.J. (2016). The sensory side of post-stroke motor rehabilitation. Restor. Neurol. Neurosci..

[23] Kasuga S., Telgen S., Uchiba J., Nozaki D., Diedrichsen J. (2015). Learning feedback and feedforward control in a mirror-reversed visual environment. J. Neurophysiol..

[24] Ackerley S.J., Stinear C.M., Barber P.A., Byblow W.D. (2014). Priming sensorimotor cortex to enhance task-specific training after subcortical stroke. Clin. Neurophysiol..

[25] Tokimura H., Di Lazzaro V., Tokimura Y., Oliviero A., Profice P., Insola A., Mazzone P., Tonali P., Rothwell J.C. (2000). Short latency inhibition of human hand motor cortex by somatosensory input from the hand. J. Physiol..

[26] Tokimura H., Ridding M.C., Tokimura Y., Amassian V.E., Rothwell J.C. (1996). Short latency facilitation between pairs of threshold magnetic stimuli applied to human motor cortex. Electroencephalogr. Clin. Neurophysiol..

[27] Koizume Y., Hirano M., Kubota S., Tanaka S., Funase K. (2017). Relationship between the changes in M1 excitability after motor learning and arousal state as assessed by short-latency afferent inhibition. Behav. Brain Res..

[28] Sawaki L., Boroojerdi B., Kaelin-Lang A., Burstein A.H., Butefisch C.M., Kopylev L., Davis B., Cohen L.G. (2002). Cholinergic influences on use-dependent plasticity. J. Neurophysiol..

[29] Meintzschel F., Ziemann U. (2006). Modification of practice-dependent plasticity in human motor cortex by neuromodulators. Cereb. Cortex.

[30] Ackerley S.J., Stinear C.M., Barber P.A., Byblow W.D. (2010). Combining theta burst stimulation with training after subcortical stroke. Stroke.

[31] Di Lazzaro V., Profice P., Pilato F., Capone F., Ranieri F., Florio L., Colosimo C., Pravatà E., Pasqualetti P., Dileone M. (2012). The level of cortical afferent inhibition in acute stroke correlates with long-term functional recovery in humans. Stroke.

[32] Brown K.E., Neva J.L., Feldman S.J., Staines W.R., Boyd L.A. (2018). Sensorimotor integration in chronic stroke: Baseline differences and response to sensory training. Restor. Neurol. Neurosci..

[33] Woodbury M.L., Velozo C.A., Richards L.G., Duncan P.W., Studenski S., Lai S.M. (2007). Dimensionality and construct validity of the Fugl-Meyer Assessment of the upper extremity. Arch. Phys. Med. Rehabil..

[34] Desrosiers J., Bravo G., Hébert R., Dutil E., Mercier L. (1994). Validation of the Box and Block Test as a measure of dexterity of elderly people: Reliability, validity, and norms studies. Arch. Phys. Med. Rehabil..

[35] Mathiowetz V., Volland G., Kashman N., Weber K. (1985). Adult norms for the Box and Block Test of manual dexterity. Am. J. Occup. Ther..

[36] Bell-Krotoski J., Tomancik E. (1987). The repeatability of testing with Semmes-Weinstein monofilaments. J. Hand Surg. Am..

[37] Brant-Zawadzki M., Gillan G.D., Nitz W.R. (1992). MP RAGE: A three-dimensional, T1-weighted, gradient-echo sequence--initial experience in the brain. Radiology.

[38] Rorden C., Brett M. (2000). Stereotaxic display of brain lesions. Behav. Neurol..

[39] Liew S.L., Schweighofer N., Cole J.H., Zavaliangos-Petropulu A., Lo B.P., Han L.K., Hahn T., Schmaal L., Donnelly M., Jeong J.N. (2023). Association of Brain Age, Lesion Volume, and Functional Outcome in Patients With Stroke. Neurology.

[40] Liew S.L., Zavaliangos-Petropulu A., Jahanshad N., Lang C.E., Haywards K.S., Lohse K.R., Juliano J.M., Assogna F., Baugh L.A., Bhattacharya A.K. (2022). The ENIGMA Stroke Recovery Working Group: Big data neuroimaging to study brain-behavior relationships after stroke. Hum. Brain Mapp..

[41] Vucic S., Chen K.H.S., Kiernan M.C., Hallett M., Benninger D.H., Di Lazarro V., Rossini P.M., Benussi A., Berardelli A., Currà A. (2023). Clinical diagnostic utility of transcranial magnetic stimulation in neurological disorders. Updated report of an IFCN committee. Clin. Neurophysiol..

[42] Rossini P.M., Burke D., Chen R., Cohen L.G., Daskalakis Z., Di Iorio R., Di Lazzaro V., Ferreri F., Fitzgerald P.B., George M.S. (2015). Non-invasive electrical and magnetic stimulation of the brain, spinal cord, roots and peripheral nerves: Basic principles and procedures for routine clinical and research application. An updated report from an IFCN Committee. Clin. Neurophysiol..

[43] Mishory A., Molnar C., Koola J., Li X., Kozel A., Myrick H., Stroud Z., Nahas Z., George M.S. (2004). The maximum-likelihood strategy for determining transcranial magnetic stimulation motor threshold, using parameter estimation by sequential testing is faster than conventional methods with similar precision. J. ECT.

[44] Butefisch C.M., Netz J., Wessling M., Seitz R.J., Hömberg V. (2003). Remote changes in cortical excitability after stroke. Brain.

[45] Toepp S.L., Turco C.V., Reshi R.S., Nelson A.J. (2021). The distribution and reliability of TMS-evoked short- and long-latency afferent interactions. PLoS ONE.

[46] Ehrenstein W.H., Ehrenstein A., Windhorst U., Johansson H. (1999). Psychophysical methods. Modern Techniques in Neuroscience Research.

[47] Fischer M., Orth M. (2011). Short-latency sensory afferent inhibition: Conditioning stimulus intensity, recording site, and effects of 1 Hz repetitive TMS. Brain Stimul..

[48] Du X., Summerfelt A., Chiappelli J., Holocomb H.H., Hong L.E. (2014). Individualized brain inhibition and excitation profile in response to paired-pulse TMS. J. Mot. Behav..

[49] Asmussen M.J., Jacobs M.F., Lee K.G.H., Zapallow C.M., Nelson A.J. (2013). Short-Latency Afferent Inhibition Modulation during Finger Movement. PLoS ONE.

[50] Turco C.V., El-Sayes J., Savoie M.J., Fassett H.J., Locke M.B., Nelson A.J. (2018). Short- and long-latency afferent inhibition; uses, mechanisms and influencing factors. Brain Stimul..

[51] Paulus W., Classen J., Cohen L.G., Large C.H., Di Lazzaro V., Nitsche M., Pascual-Leone A., Rosenow F., Rothwell J.C., Ziemann U. (2008). State of the art: Pharmacologic effects on cortical excitability measures tested by transcranial magnetic stimulation. Brain Stimul..

[52] Dobkin B.H. (2003). Do electrically stimulated sensory inputs and movements lead to long-term plasticity and rehabilitation gains?. Curr. Opin. Neurol..

[53] Mirdamadi J.L., Block H.J. (2020). Somatosensory changes associated with motor skill learning. J. Neurophysiol..

[54] Honaga K., Fujiwara T., Tsuji T., Hase K., Uchiba J., Liu M. (2013). State of intracortical inhibitory interneuron activity in patients with chronic stroke. Clin. Neurophysiol..

[55] Turco C.V., Locke M.B., El—Sayes J., Tommerdahl M., Nelson A.J. (2018). Exploring behavioral correlates of afferent inhibition. Brain Sci..

[56] Baker A., Schranz C., Seo N.J. (2023). Associating Functional Neural Connectivity and Specific Aspects of Sensorimotor Control in Chronic Stroke. Sensors.

[57] Schranz C., Srivastava S., Seamon B.A., Marebwa B., Bonilha L., Ramakrishnan V., Wilmskoetter J., Neptune R.R., Kautz S.A., Seo N.J. (2023). Different aspects of hand grip performance associated with structural connectivity of distinct sensorimotor networks in chronic stroke. Physiol. Rep..

[58] Oliviero A., Molina Leon A., Holler I., Florensa Vila J., Siebner H.R., Della Marca G., Di Lazzaro V., Tejeira Alvarez J. (2005). Reduced sensorimotor inhibition in the ipsilesional motor cortex in a patient with chronic stroke of the paramedian thalamus. Clin. Neurophysiol..

[59] Alaydin H.C., Ataoglu E.E., Caglayan H.Z.B., Togoz N., Cengiz B. (2021). Short-latency afferent inhibition remains intact without cortical somatosensory input: Evidence from a patient with isolated thalamic infarct. Brain Stimul. Basic Transl. Clin. Res. Neuromodul..

[60] Vidoni E.D., Boyd L.A. (2009). Preserved motor learning after stroke is related to the degree of proprioceptive deficit. Behav. Brain Funct..

[61] Blaschke J., Vatinno A.A., Scronce G., Ramakrishnan V., Seo N.J. (2022). Effect of Sensory Impairment on Hand Functional Improvement with Therapy and Sensory Stimulation. Neurol. Neurorehabilit..

[62] Tyson S.F., Hanley M., Chillala J., Selley A.B., Tallis R.C. (2008). Sensory loss in hospital-admitted people with stroke: Characteristics, associated factors, and relationship with function. Neurorehabilit. Neural Repair.

[63] Meyer S., Karttunen A.H., Thijs V., Feys H., Verheyden G. (2014). How do somatosensory deficits in the arm and hand relate to upper limb impairment, activity, and participation problems after stroke? A systematic review. Phys. Ther..

[64] Hammerbeck U., Tyson S.F., Samraj P., Hollands K., Krakauer J.W., Rothwell J. (2021). The Strength of the Corticospinal Tract Not the Reticulospinal Tract Determines Upper-Limb Impairment Level and Capacity for Skill-Acquisition in the Sub-Acute Post-Stroke Period. Neurorehabilit. Neural Repair.

[65] Hammerbeck U., Hoad D., Greenwood R., Rothwell J. (2019). The unsolved role of heightened connectivity from the unaffected hemisphere to paretic arm muscles in chronic stroke. Clin. Neurophysiol..

[66] Buetefisch C.M., Revill K.P., Haut M.W., Kowalski G.M., Wischnewski M., Pifer M., Belagaje S.R., Nahab F., Cobia D.J., Hu X. (2018). Abnormally reduced primary motor cortex output is related to impaired hand function in chronic stroke. J. Neurophysiol..

[67] Maier M., Ballester B.R., Verschure P.F.M.J. (2019). Principles of Neurorehabilitation after Stroke Based on Motor Learning and Brain Plasticity Mechanisms. Front. Syst. Neurosci..

[68] Fisicaro F., Lanza G., Grasso A.A., Pennsisi G., Bella R., Paulus W., Pennisi M. (2019). Repetitive transcranial magnetic stimulation in stroke rehabilitation: Review of the current evidence and pitfalls. Ther. Adv. Neurol. Disord..

[69] Edwards L.L., King E.M., Buetefisch C.M., Borich M.R. (2019). Putting the “sensory” into sensorimotor control: The role of sensorimotor integration in goal-directed hand movements after stroke. Front. Integr. Neurosci..

[70] Seo N.J., Kamper D.G., Ramakrishnan V., Harvey J.B., Finetto C., Schranz C., Scronce G., Coupland K., Howard K., Blaschke J. (2022). Effect of novel training to normalize altered finger force direction post-stroke: Study protocol for a double-blind randomized controlled trial. Trials.

[71] Palmer J.A., Wolf S.L., Borich M.R. (2018). Paired associative stimulation modulates corticomotor excitability in chronic stroke: A preliminary investigation. Restor. Neurol. Neurosci..

[72] Turco C.V., Pesevski A., McNicholas P.D., Beaulieu L.D., Nelson A.J. (2019). Reliability of transcranial magnetic stimulation measures of afferent inhibition. Brain Res..

[73] Brown K.E., Lohse K.R., Mayer I.M.S., Stigaro G., Desikan M., Capsula E.P., Meunier S., Popa T., Lahmy J.-C., Odish O. (2017). The reliability of commonly used electrophysiology measures. Brain Stimul..

[74] Wang L., Wang S., Zhang S., Dou Z., Guo T. (2023). Effectiveness and electrophysiological mechanisms of focal vibration on upper limb motor dysfunction in patients with subacute stroke: A randomized controlled trial. Brain Res..

[75] Kutch J.J., Valero-Cuevas F.J. (2011). Muscle redundancy does not imply robustness to muscle dysfunction. J. Biomech..

[76] Cruz E.G., Waldinger H.C., Kamper D.G. (2005). Kinetic and kinematic workspaces of the index finger following stroke. Brain.

[77] Kamper D., Barry A., Bansal N., Stoykov M.E., Triandafilou K., Vindakovic L., Seo N.J., Roth E. (2022). Use of cyproheptadine hydrochloride (HCl) to reduce neuromuscular hypertonicity in stroke survivors: A Randomized Trial: Reducing Hypertonicity in Stroke. J. Stroke Cerebrovasc. Dis..

[78] Ruddy K.L., Jaspers E., Keller M., Wenderoth N. (2016). Interhemispheric sensorimotor integration; an upper limb phenomenon?. Neuroscience.

[79] Smith M.C., Ackerley S.J., Barber P.A., Byblow W.D., Stinear C.M. (2019). PREP2 Algorithm Predictions Are Correct at 2 Years Poststroke for Most Patients. Neurorehabilit. Neural Repair.

